# The Effect of Novel Support Layer by Titanium-Modified Plasma Nitriding on the Performance of AlCrN Coating

**DOI:** 10.3390/ma18174186

**Published:** 2025-09-06

**Authors:** Jiqiang Wu, Longchen Zhao, Jianbin Ji, Fei Sun, Jing Hu, Xilang Liu, Dandan Wang, Xulong An, Xiangkui Liu, Wei Wei

**Affiliations:** 1 Jiangsu Province Key Laboratory of Materials Surface Science and Technology, Changzhou University, Changzhou 213164, Chinaxulong@126.com (X.A.); xiangkui@126.com (X.L.); benjamin.wwei@163.com (W.W.); 2 Huaide College, Changzhou University, Jingjiang 214500, China; 3 Department of Modern Equipment Manufacturing, Changzhou Institute of Industry Technology, Changzhou 213164, China

**Keywords:** plasma nitriding, compound layer, AlCrN coating, composite treatment, adhesion strength, performances

## Abstract

In order to obtain a gradient coating with excellent performance, novel titanium-modified plasma nitriding was primarily used as a support layer for the PVD coating of 38CrMoAl steel. The samples were subjected to titanium-modified plasma nitriding by placing sponge titanium around the samples, resulting in a thicker ductile diffusion layer and a thinner and denser compound layer. The research results showed that this thinner, denser compound layer formed by titanium-modified plasma nitriding provides stronger support for the AlCrN coating and thus bring about better performance compared to a conventional plasma nitrided layer, with the adhesion strength increasing from 16.8 N to 29.4 N, which is 42.8% higher than the conventional PN compound layer; the surface hardness increasing from 3650 HV_0.05_ to 3780 HV_0.05_; the friction coefficient and wear rate reducing from 0.64 and 5.4849 × 10^−6^ mm^3^/(N·m) to 0.61 and 2.3060 × 10^−6^ mm^3^/(N·m), respectively; and the wear performance improving by 137.85%. Additionally, the corrosion potential increased from −979.2 mV to −711.51 mV, and the value of impedance increased from 1.5515 × 10^4^ Ω·cm^2^ to 9.4518 × 10^4^ Ω·cm^2^, resulting in a significant improvement in corrosion resistance. In all, the novel support layer by titanium-modified plasma nitriding can provide much better support for AlCrN coating and thus bring about excellent enhanced performances, including adhesion strength and wear and corrosion resistance. Therefore, it is of great value in the PVD coating field, and it can provide valuable insights into gradient coating technology.

## 1. Introduction

38CrMoAl steel has a good combination of strength and toughness, and is widely used in molds and cutting tools, commonly surface-hardened by plasma nitriding to enhance its surface properties to meet the technical requirements of the components [1,2,3]. However, with increasingly harsh service conditions, the nitriding layer is prone to losing effectiveness due to wear and erosion or premature cracking and shedding of the compound layer. Therefore, it is necessary to obtain a gradient composite coating with excellent performance in order to meet the technical requirements for components subjected to harsh service conditions.

Physical vapor deposition (PVD) coatings such as AlCrN, TiAlN, and CrTiAlN have advantages such as high hardness and excellent wear and corrosion resistance [4,5], which can effectively protect steel components from premature failure resulting from surface wear and corrosion. However, if a single-layered coating is directly deposited onto the surface of a softer steel, an “eggshell effect” will occur, leading to premature failure of the coating. In order to make good use of the advantages of PVD coatings and avoid premature crack failure, pre-treatment has been introduced to improve their performance, which can not only increase the load-bearing capacity of PVD coatings but also improve fatigue strength and wear and corrosion resistance.

It has been reported that plasma nitriding is a useful pre-treatment for PVD coatings that can increase load-bearing capacity and improve fatigue strength and wear resistance [6,7,8]. While divergent views exist regarding the influence of the nitrided layer on the performance of PVD coatings, some studies suggest that the nitrided layer is beneficial [9], whereas others indicate that the nitrided layer affects the performance of PVD coatings [10].

Based on our previous research [11,12], the addition of titanium can effectively modify the characteristics of the nitrided layer, and thus, a plasma nitrided layer with a thicker ductile diffusion layer, thinner brittle compound layer, and higher surface hardness can be obtained. Titanium can significantly decrease the compound layer from 19 μm to 10 μm and increase the effective hardening layer, and also greatly enhance the surface hardness from 703 HV_0.05_ to 889 HV_0.05_. More importantly, both wear resistance and toughness can be greatly improved by the novel titanium-modified plasma nitrided layer, attributed to the formation of dispersed TiN particles with very high hardness in the nitriding layer. Therefore, it is valuable to clarify how the nitrided layer affects the performance of PVD coatings. To this end, innovative titanium-modified plasma nitriding was primarily adopted as a pre-treatment prior to the deposition of AlCrN coating in this research, since it has been reported that a thinner, denser compound layer and thicker effective hardening layer can be formed by this novel titanium-enhanced plasma nitriding [11,12]. The goal of this research is to obtain a gradient composite coating with excellent performance by regulating the characteristics of the nitriding layer, especially with a denser structure and a lower ratio of compound layer to effective hardening layer thickness, and to analyze the related mechanisms as well.

## 2. Materials and Methods

38CrMoAl steel with a hardness of 320–350 HV_0.05_ was used in this research; its chemical composition (wt.%) is as follows: C: 0.38; Cr: 1.51, Mo: 0.20; Mn: 0.50; Si: 0.30; Al: 0.95; S: 0.012, and Fe: balance. The samples were processed into a size of 10 mm*10 mm*8 mm, then ground using 240–2000 mesh emery papers to obtain a surface roughness less than Ra0.4 μm, and finally, ultrasonically cleaned prior to use.

Titanium-modified plasma nitriding (PNTi) was carried out at a temperature of 500 °C for 4 h in a mixture gas of N_2_ + H_2_, with the ratio of N_2_:H_2_ being 1:3 and with a gas pressure of 300 Pa [13], followed by furnace cooling. A few titanium particles with a diameter of about 3~5 mm were placed around the samples for plasma nitriding, and the number of titanium particles was 10 mg per square millimeter of the samples’ surface area, i.e., 10 mg/mm^2^. Meanwhile, traditional plasma nitriding (PN) with the same conditions, including temperature, process duration time, mixture gas, and pressure, was also conducted as a reference for a comparative study. After PN and PNTi, the AlCrN coatings were deposited by an ion plating equipment (Xinghu, Suzhou, China) at a temperature of 420 °C for 5 h in an atmosphere of N_2_+Ar, with the ratio of N_2_:Ar being 1:3 and with a gas pressure of 3 Pa, and with a CrN layer as a transition layer. The parameters for plasma nitriding and AlCrN coatings deposition are shown in Table 1 and Table 2.

The surface morphology and cross-sectional microstructure of the treated samples were observed by a JSM-IT100 (Leica Microsystems, Wetzlar, Germany) scanning electron microscope (SEM) and a DMI-3000M optical microscope (Huahui, Lanzhou, China). The surface roughness of the treated samples was observed by Atomic Force Microscopy Dimenson ICON (Bruker Corporation, Billerica, MA, USA); the scanning area range is 5 μm × 5 μm. Phase constituents were determined by D/max2500 X-ray diffraction (XRD) with Cu-Ka radiation. The microstructures of the coatings were determined using a transmission electron microscope (HRTEM, FEI-F20, Tecnai, Stanford, CA, USA). The cross-sectional microstructure of the coating was observed after preparing the specimen with end-milling by a focus ion beam (FIB) system operated at 4.5 × 10^3^ eV and an incident angle of 3–8°. The cross-sectional microhardness was measured by the HXD-1000 TMC Vickers microhardness tester (Oupu, Changzhou, China) with a load of 50 g for 15 s, and the surface hardness was measured by Nano Indenter G200, with the penetration depth being 1/10 of the AlCrN coating thickness. Subsequently, the adhesion strength between the coating and the substrate was determined by multi-functional MFT-4000 using a continuously increasing load on a 200 μm radius diamond tip producing a scratch at a sliding speed of 30 N/min. Meanwhile, the wear resistance of the samples was tested using a pin-on-disc tribometer by multi-functional MFT-4000 (Huahui, Lanzhou, China) under conditions of dry sliding against a 3.0 mm diameter SiC hard metal ball, at a linear speed of 220 mm/min, with a normal load of 20 N for 30 min. Finally, the wear track profiles of samples after the wear test were determined by a laser spectroscopy confocal microscope. The corrosion properties of the samples were analyzed using a Gamry electrochemical workstation (Runsi, Zhengzhou, China).

## 3. Results and Discussion

### 3.1. Cross-Sectional Microstructures

**Figure 1:**
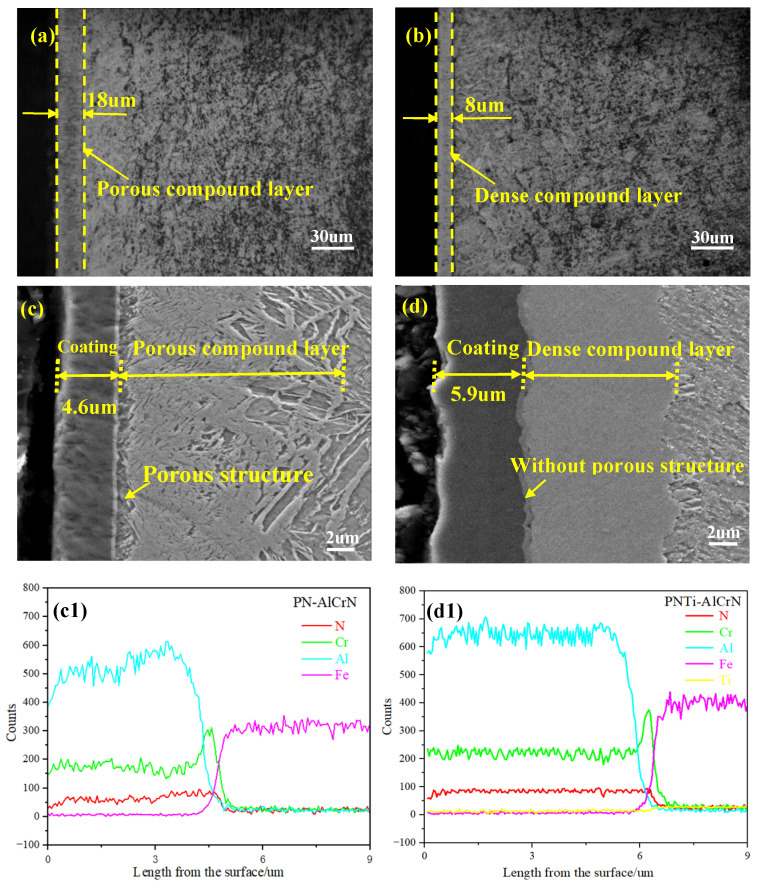
Cross-sectional microstructures and EDS of various coatings. (**a**) PN, (**b**) PNTi, (**c**) PN + AlCrN, (**d**) PNTi + AlCrN, (**c**,**d**) cross-sectional EDS line scanning for N, Cr, and Al corresponding to samples in (**c1**,**d1**).

Figure 1a–d displays the cross-sectional microstructures of various coatings. As shown in Figure 1a,b, the thicknesses of the compound layer by PNTi and conventional PN are 8 μm and 18 μm, respectively. In addition, it can be observed that the compound layer obtained by PNTi is denser than that by conventional PN. Notably, the thicknesses of the AlCrN coating are 4.6 μm and 5.9 μm in Figure 1c,d, respectively, i.e., the AlCrN coating with a pre-treatment of PNTi is obviously thicker. Meanwhile, it can be seen that the connection location between the conventional PN compound layer and the AlCrN coating presents a porous structure, while the connection location between the titanium-enhanced PN compound layer and the AlCrN coating is dense without a porous structure. The cross-sectional EDS line scanning for N, Cr, and Al corresponding to samples in Figure 1c,d is shown in Figure 1(c1,d1); it can be seen that the N, Cr, and Al element concentration in the thin compound layer is smoother than that in the thick compound layer. By comparison, it can be seen that the thin and dense compound layer obtained by titanium-enhanced PN is more conducive to the growth of the AlCrN coating than the conventional porous compound layer under the same coating depositing process. These results demonstrate that the thin and dense compound layer obtained by PNTi is more conducive to the growth of the AlCrN coating than the conventional porous compound layer under the same coating process. Based on the above research results, it can be inferred that the adhesion strength of the AlCrN coating and compound layer may be enhanced.

### 3.2. Surface Roughness

**Figure 2:**
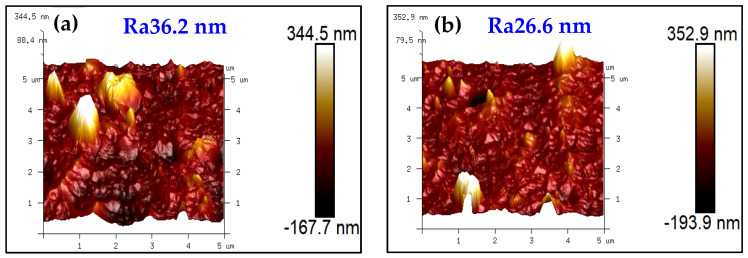
AFM surface morphology of various coatings. (**a**) PN-AlCrN, (**b**) PNTi-AlCrN.

Figure 2a,b shows that the surface roughness is Ra36.2 nm and Ra26.6 nm for the complex coatings with the PN layer and the PNTi layer, respectively, indicating a significant decrease of 26.5% in surface roughness by the PNTi layer compared to the PN layer. It further demonstrates that a thin and dense PNTi compound layer helps reduce the surface roughness of the coating.

### 3.3. XRD Analysis

**Figure 3:**
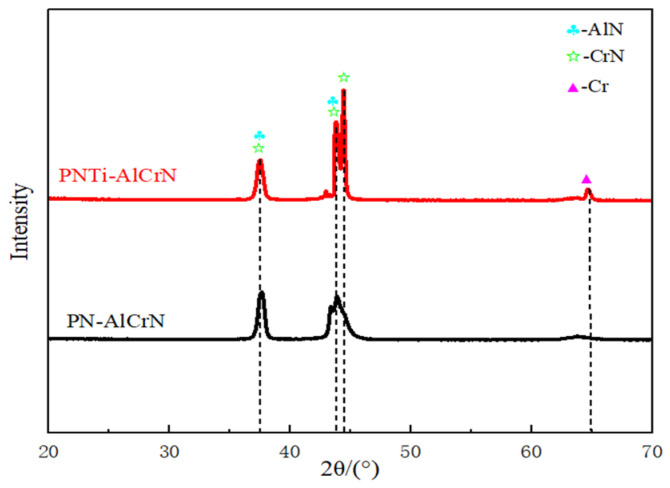
X-ray diffraction patterns of various coatings.

Figure 3 shows the X-ray diffraction patterns of various coatings. It can be seen that although the main phase in both cases is (Cr,Al)N when AlCrN coatings are deposited onto PN or PNTi layers, the diffraction intensity is stronger in the multilayer structure combining the AlCrN coating with the PNTi layer. And it needs to be noted that the main phases of Fe_2–3_N and Fe_4_N in the PN layer [1] disappear in the complex layer.

### 3.4. Hardness Distribution

**Figure 4:**
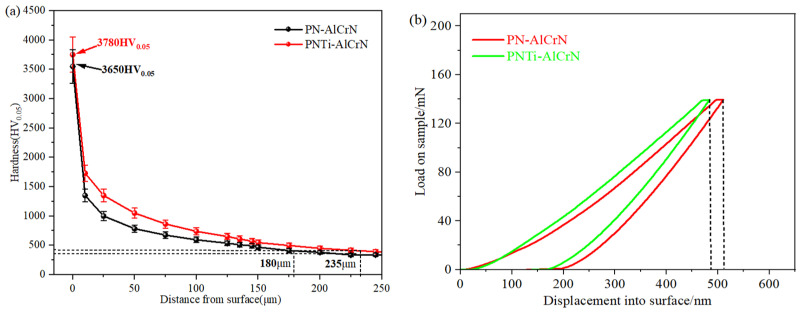
Cross-sectional microhardness of various coatings. (**a**) Hardness gradient curve, (**b**) nanoindentation curve.

The hardness value is the average of five measurements taken. The indentation depth is less than 1/10 of the coating thickness according to the nanoindentation curve in Figure 4b, indicating the validity of the indentation experimental results. It can be seen that surface hardness of approximately 3780 HV_0.05_ is obtained by the PNTi layer combined with the AlCrN coating, significantly higher than that of 3650 HV_0.05_ with the conventional PN layer in Figure 4a; it is approximately four times the surface hardness of the PNTi layer [11,12], which may be attributed to the better support from the denser PN compound layer. Meanwhile, the hardness gradient of the complex coating with the PNTi layer is gentler than that with the traditional PN layer, and the effective hardening layer increases from 180 μm to 235 μm. The increase in surface hardness and effective hardening layer is due to the diffusion of alloying elements into the PN layer and formation of hard nitrided phases such as TiN and (Cr,Al)N during the deposition of the AlCrN coating, which is consistent with the results of the coating phases in Section 3.3.

### 3.5. Adhesion Strength

**Figure 5:**
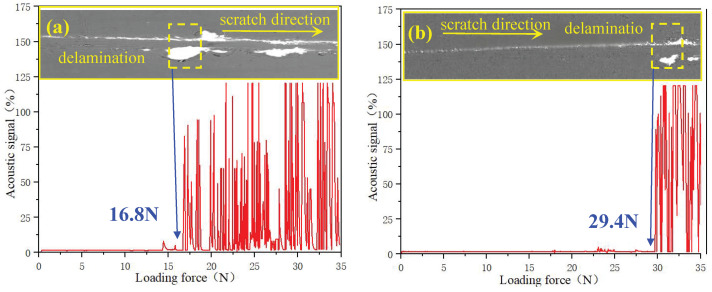
Acoustic emission signal and scratch track morphology of various coatings. (**a**) PN-AlCrN, (**b**) PNTi-AlCrN.

Figure 5a,b shows that the critical loads of specimens are 16.8 N and 29.4 N for the complex coatings with the PN layer and the PNTi layer, respectively, demonstrating a significant increase of 42.8% in adhesion strength by the PNTi layer compared to the PN layer, which is consistent with the cross-sectional microstructures of coatings in Section 3.1.

### 3.6. Wear Tests

In order to evaluate the differences in wear resistance among various coatings, the specific wear rate calculation method, W = V/(NL) [7], was adopted for quantitative comparative analysis, where V represents the volume loss difference of the workpiece before and after wear (mm^3^), N is the applied load (N), L is the sliding distance (m). The wear depth, width, and wear rate of the wear test are the average values of three measurements.

**Figure 6:**
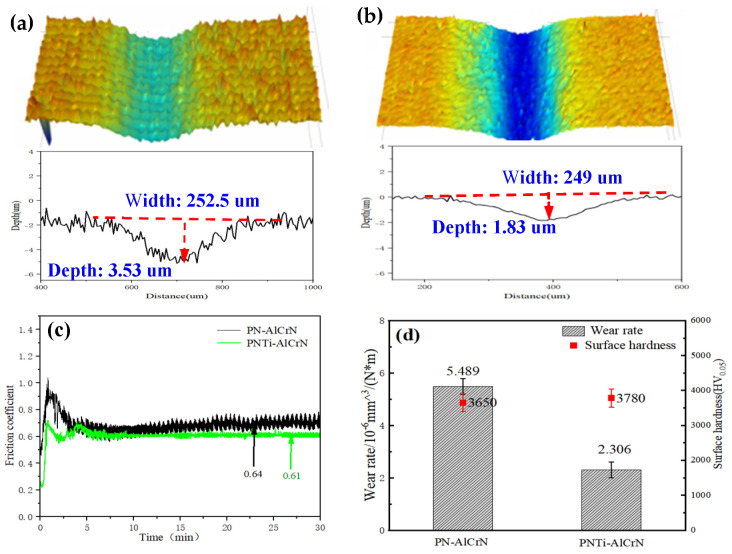
Wear profiles and performance for various coatings. (**a**) Wear profile: PN-AlCrN, (**b**) wear profile: PNTi-AlCrN, (**c**) friction coefficient, (**d**) wear rate and surface hardness.

Figure 6 displays the wear track profiles and the friction coefficient of complex coatings after the wear test. It can be clearly seen the wear depth of samples by complex coating with PNTi is only 1.83 μm in Figure 6b, significantly lower than that of 3.53 μm by conventional PN in Figure 6a; the average friction coefficient of the PNTi complex coating is 0.61, and the wear rate is 2.306 × 10^−6^ mm^3^/(N·m), while the average friction coefficient of the PN complex coating is 0.64. The wear rate is 5.489 × 10^−6^ mm^3^/(N·m) in Figure 6c,d, and the wear performance has improved by 137.85%, demonstrating that PNTi can bring about better wear resistance.

### 3.7. Corrosion Tests

**Figure 7:**
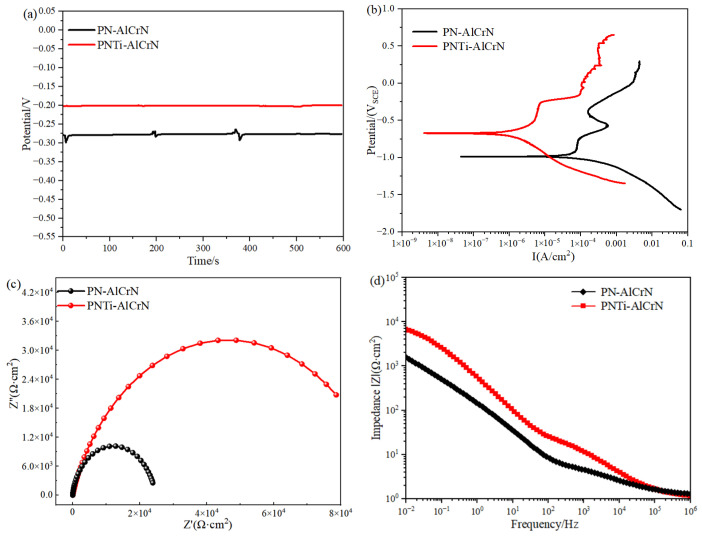
Corrosion resistance test results of various coatings in 3.5% NaCl solution. (**a**) Open-circuit potential, (**b**) polarization curves, (**c**) Nyquist plots, (**d**) Bode plots.

Figure 7 and Table 3 illustrate the corrosion resistance test results of various duplex coatings in 3.5% NaCl solution. The open-circuit potential values of complex coatings finally stabilized with time in Figure 7a. Compared to the PN complex coating, the corrosion potential of the PNTi complex coating increased from −979.2 mV to −711.51 mV, and the corrosion current decreased from 0.70701 × 10^−5^ A/cm^2^ to 0.06445 × 10^−5^ A/cm^2^. According to previous studies [14], a larger capacitive arc radius indicates better corrosion resistance, and the PNTi complex coating displays a larger arc radius in Figure 7c; the value of impedance increased from 1.5515 × 10^4^ Ω·cm^2^ to 9.4518 × 10^4^ Ω·cm^2^ in Table 3, signifying a higher polarization resistance and indicating better corrosion resistance.

**Figure 8:**
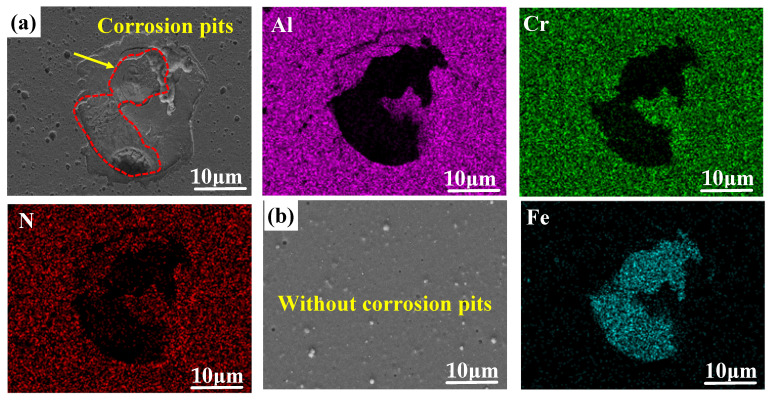
Surface corrosion morphology and EDS results after electrochemical test of various coatings. (**a**) PN-AlCrN, (**b**) PNTi-AlCrN.

In addition, it can be clearly seen that there are large areas of pitting corrosion pits on the surface of the coating in Figure 8a; the main elements Al, Cr, and N of the coating have corroded and disappeared, presenting the Fe element in the steel matrix. Meanwhile, there are no pitting corrosion pits in Figure 8b, proving the good corrosion resistance of the PNTi composite coating.

## 4. Mechanisms Analysis

**Figure 9:**
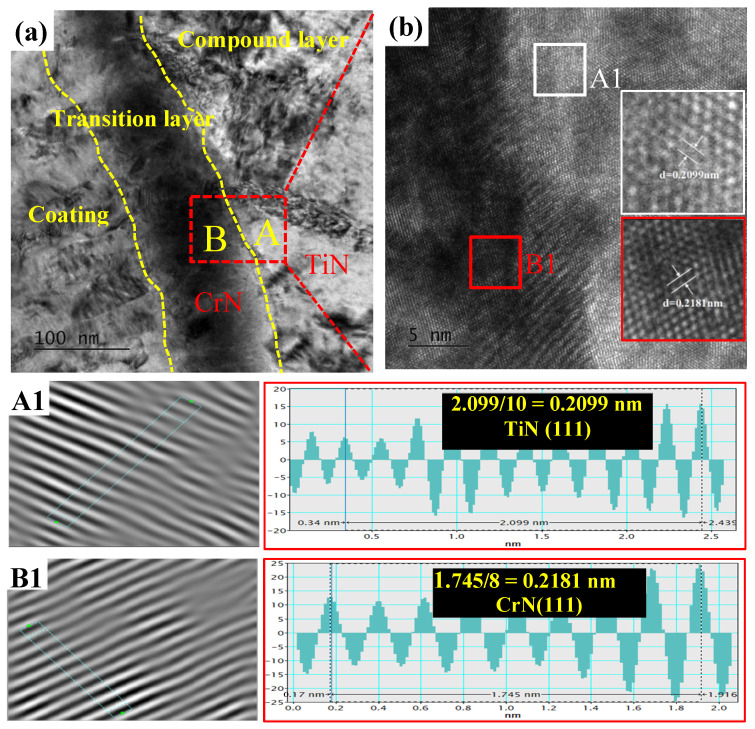
Microstructure at the interface of PNTi layer and AlCrN coatings. (**a**) HAADF image, (**b**) HRTEM image; (**A1**,**B1**) IFFT diagrams of the area (A) and (B).

Based on the above results, the complex coating produced by combining titanium-enhanced PN with AlCrN deposition exhibits superior adhesion and performance. This enhancement is primarily attributable to the thin and dense compound layer containing a hard TiN phase [11] formed during the titanium-modified PN process (Figure 1). Firstly, the TiN phase formed in the PNTi compound layer can serve as additional nucleation centers for the formation of the CrN phase in the transition layer, resulting in a decrease in grain size and an increase in the growth rate of the coating layer, which can enhance the hardness and coating layer thickness. The HRTEM image of the interface between the dense PNTi compound layer (Zone A) and the transition layer (Zone B) of CrN coating is shown in Figure 9. By performing inverse IFFT imaging, the lattice spacing of the CrN transition layer and the TiN in the PNTi compound layer was calculated to be 0.2181 nm and 0.2099 nm, respectively. It can be determined by the Turnbull Vonnegut formula [15] whether the CrN transition layer and the TiN in the PNTi compound layer are coherent:

(1) $$\delta =\frac{2\left|d_{\alpha }-d_{\beta }\right|}{d_{\alpha }+d_{\beta }}$$

The lattice mismatch between the CrN transition layer and the TiN in the PNTi compound layer was calculated to be 3.83% by Formula (1); the value is less than 5%, indicating that the bonding interface between the CrN transition layer and the TiN in the PNTi compound layer is completely coherent [15,16,17], enabling epitaxial growth of the coating on the compound layer. This structural coherence accelerates coating deposition rates and strengthens interfacial adhesion between the coating and substrate.

## 5. Conclusions

The thinner and denser compound layer formed by PNTi provides stronger support for the AlCrN coating compared to conventional PN, which can effectively increase the surface hardness of the coating from 3650 HV_0.05_ to 3780 HV_0.05_.

The bonding interface between the CrN transition layer and the TiN in the PNTi compound layer is completely coherent, which is conducive to the epitaxial diffusion growth of the coating, increasing the adhesion strength from 16.8 N to 29.4 N, which is 42.8% higher than the conventional PN compound layer.

During the novel duplex treatment via titanium-enhanced plasma nitriding and deposition of AlCrN coating, the effective hardening layer increased from 180 μm to 235 μm, resulting in the wear rate decreasing from 5.4849 × 10^−6^ mm^3^/(N·m) to 2.3060 × 10^−6^ mm^3^/(N·m), i.e., the wear performance improved by 137.85%. Furthermore, the corrosion potential of the PNTi complex coating increased from −979.2 mV to −711.51 mV, demonstrating better wear and corrosion resistance.

## Figures and Tables

**Table 1:** Parameters for plasma nitriding.

Treatment	Plasma Nitriding				
	Atmosphere	Titanium (g)	Temperature (°C)	Time (h)	Pressure (Pa)
PN	N_2_:H_2_ = 1:3	Without	500	4	300
PNTi	N_2_:H_2_ = 1:3	0.8			

**Table 2:** Parameters for plasma nitriding and PVD coating deposition.

Treatment	Plasma Nitriding	AlCrN Coating Deposition					
	/	Atmosphere	Temperature (°C)	Time (h)	Pressure (Pa)	Bias Voltage (V)	Current (A)
PN-AlCrN	Table 1	N_2_:Ar = 1:3	420	5	3	−80	75
PNTi-AlCrN							

**Table 3:** Corrosion resistance test results of various coatings in 3.5% NaCl solution.

Sample	Corrosion Performance Parameters		
	E_corr_/mV	I_corr_/10^−5^ A/cm^2^	R_P_/10^4^ Ω·cm^2^
PN-AlCrN	−979.2	0.70701	1.5515
PNTi-AlCrN	−711.51	0.06445	9.4518

## Data Availability

The original contributions presented in this study are included in the article. Further inquiries can be directed to the corresponding author.
